# High-risk factors for postoperative complications in patients with pancreatic disease: a single-center experience

**DOI:** 10.3389/fsurg.2025.1689107

**Published:** 2025-11-19

**Authors:** Man Zhang, Ning Li, Wenjie Tian, Qigui Xiao, Kongyuan Wei, Zheng Wang, Huapeng Lu

**Affiliations:** Department of Hepatobiliary and Pancreas Surgery, The First Affiliated Hospital of Xi'an JiaoTong University, Xian, China

**Keywords:** hospitalisation, pancreatic fistula, pancreatic disease, nutritional status, hospital complications, pancreatoduodenectomy, pancreatectomy

## Abstract

**Objective:**

To collect and analyse the hospital complication status of hospitalised patients with pancreatic disease in a tertiary hospital in western China and to explore the influencing factors, providing a foundation for further research.

**Methods:**

A retrospective study design was adopted. Electronic medical records of pancreatic surgery patients hospitalised at the First Affiliated Hospital of Xi'an JiaoTong University from March 1, 2024, to July 31, 2024, were retrospectively reviewed. Data on demographic characteristics, NRS2002 scores, diagnoses, laboratory results, surgical methods, and complications were collected. SPSS software was used for univariate and multivariate analyses.

**Results:**

In total, 172 patients, with a mean age of 60.21 ± 10.94 years, were included. Hospital complications occurred in 21.51% of pancreatic disease patients. The three most common complications were infection (14.53%), pancreatic fistula (7.56%), and cholangitis (3.49%). Univariate analysis revealed that disease diagnosis category (*χ*² = 8.342, *P* = 0.015), postoperative red blood cell (RBC) count (*t* = −2.552, *P* = 0.012), and postoperative haemoglobin concentration (Hb, g/L) (*t* = −2.393, *P* = 0.018) were risk factors for complications. Multivariate analysis confirmed that a high NRS2002 score was an independent risk factor (OR = 4.20; 95% CI: 1.017–17.368; *P* = 0.047).

**Conclusion:**

The hospital complication rate in pancreatic disease patients was 21.51%, with infection, pancreatic fistula, and cholangitis being the most common complications. Low postoperative RBC counts, low postoperative Hb concentrations, and high preoperative NRS2002 scores were significant risk factors. These findings underscore the potential clinical importance of integrated perioperative nutritional support and anaemia management in improving surgical outcomes for pancreatic tumour patients, warranting further investigations in larger prospective studies.

## 1 Introduction

Pancreatic neoplasms are highly malignant and have shown a slowly increasing incidence since 2000. Global cancer statistics indicate that approximately 490,000 individuals were diagnosed with pancreatic neoplasms in 2020, with 460,000 deaths worldwide (1). In China, the National Cancer Center estimated 76,030 new cases and 68,222 deaths in 2024 (2).

Managing pancreatic disease typically involves comprehensive treatment centred on surgical resection (3). Surgery remains the cornerstone of curative treatment; however, postoperative complications frequently occur, adversely affecting patient recovery, survival, and health care costs. Approximately 50% of hospitalised pancreatic disease patients experience hospital complications (4). A study by Weinberg et al. revealed that the median (IQR) hospital cost in US dollars was 31.6% greater for patients who experienced complications than for those who experienced no complications [40,717.8 (27,358.0–59,834.3) vs. 30,946.9 (23,910.8–46,828.1)] (5).

While previous studies have identified various risk factors, there is a paucity of data comprehensively evaluating the role of dynamic perioperative laboratory values (e.g., immediate postoperative haemoglobin levels) alongside validated nutritional screening tools (such as NRS2002) in specific regional populations, such as those in western China.

A precise understanding of these modifiable risk factors is crucial for developing targeted preventive strategies. Therefore, this study investigated postoperative complication status and influencing factors in pancreatic disease patients at a tertiary hospital in western China.

## 2 Materials and methods

### 2.1 Study design

The study adopted a retrospective study design.

### 2.2 Study population

Electronic medical records of hospitalised patients in the Department of Pancreatic Surgery at the First Affiliated Hospital of Xi'an JiaoTong University from March 1, 2024, to July 31, 2024, were retrospectively reviewed. Inclusion criteria: Adult patients aged ≥18 years. Exclusion criteria: Patients who voluntarily discharged or transferred during hospitalisation or those whose electronic records were incomplete.

### 2.3 Sample size calculation

A convenience sampling method was employed to review all eligible electronic medical records during the study period.

### 2.4 Data collection

The following data were collected: (1) Demographic characteristics: sex, age, weight, and height. (2) NRS2002 scores and diagnoses (pancreatic neoplasms, periampullary tumour, cholangiocarcinoma). (3) Preoperative laboratory results: White blood cell count (WBC, 10^9^/L), red blood cell count (RBC, 10^12^/L), haemoglobin concentration (Hb, g/L), platelet count (10^9^/L), neutrophil percentage (%), AST concentration (U/L), ALT concentration (U/L), ALP concentration (U/L), total bilirubin concentration (μmol/L), direct bilirubin concentration (μmol/L), total protein concentration (g/L), and albumin concentration (g/L). Postoperative laboratory results: WBC count, RBC count, Hb concentration, platelet count, neutrophil percentage, AST concentration, ALT concentration, ALP concentration, total bilirubin concentration, direct bilirubin concentration, total protein concentration, and albumin concentration. (4) Surgical methods: Pancreatoduodenectomy, distal pancreatectomy, endoscopic procedures, biopsy (other), or no surgery. (5) Complication status.

### 2.5 Data quality control

Two researchers independently extracted data from the electronic medical records. After data collection, discrepancies were resolved by cross-checking and reverifying against the original records. A third researcher addressed missing values and outliers by reexamining the electronic records. Although the data analysis has a cross-sectional component in assessing the incidence of complications during a specific period, the fundamental design for data collection was retrospective.

### 2.6 Statistical analysis

Data were double-entered using EpiData 3.1 to construct a database. Inconsistent entries were corrected by referencing original records, followed by logical error checks. Statistical analyses were performed with SPSS 23.0: Categorical data are expressed as percentages and were analysed using chi-square tests. Continuous data are expressed as the means ± standard deviations and were analysed using t tests, ANOVA, or SNK-q tests.

Multivariate analysis was performed using binary logistic regression to identify independent factors associated with postoperative complications (coded as Yes = 1, No = 0). We employed a forced entry approach, including all the variables collected for the study in the final model, regardless of their significance in the univariate analysis. This conservative strategy was chosen to ensure a comprehensive assessment and to minimise the risk of overlooking potential predictors. Prior to model fitting, multicollinearity among all the independent variables was assessed using the variance inflation factor (VIF). All the VIF values were less than 5, indicating no substantial multicollinearity, thus justifying their simultaneous inclusion in the model. The results are expressed as adjusted odds ratios (ORs) with 95% confidence intervals (CIs).

Categorical variables (e.g., sex, disease diagnosis, surgical procedure) were handled using dummy coding in the multivariate logistic regression model. For each categorical variable, a reference group was specified to facilitate the interpretation of odds ratios. A complete list of variable categories and their corresponding reference groups is provided in the footnotes of Table 3.

All the statistical tests were two-sided, with a significance level of *α* = 0.05.

## 3 Results

### 3.1 Baseline characteristics of included subjects

A total of 199 electronic medical records were reviewed. After 27 patients whose records were incomplete were excluded, 172 patients were included, yielding an effective sample size of 172 patients. Among them, 101 were men and 71 were women, with ages ranging from 26 to 82 years and a mean age of 60.21 ± 10.94 years (Table 2).

### 3.2 Hospital complications in pancreatic disease patients

Among the 172 patients, 37 (21.51%) experienced postoperative complications. The most common complication was infection (25 cases, 14.53%), followed by pancreatic fistula (13 cases, 7.56%) and cholangitis (6 cases, 3.49%). The complete spectrum of complications is detailed in Table 1.

**Table 1:** Hospital complications in patients with pancreatic disease.

Complication	*N* (%)
Infection	25 (14.53)
Pancreatic fistula	13 (7.56)
Cholangitis - postoperative inflammatory obstruction of the lower biliary tract	6 (3.49)
Cholestatic hepatitis	6 (3.49)
Ascites	6 (3.49)
Post-ERCP pancreatitis	3 (1.74)
Pleural effusion	3 (1.74)
Abdominal bleeding	3 (1.74)
Biliary fistula	2 (1.16)
Electrolyte disturbance	2 (1.16)
Diarrhoea with bloating	2 (1.16)
Respiratory failure	2 (1.16)
Jaundice/ obstructive jaundice	2 (1.16)
Chylous fistula	2 (1.16)
Inflammatory reaction	2 (1.16)
Peripheral vascular and venous thrombosis	2 (1.16)
Intestinal fistula	1 (0.58)
Pulmonary embolism	1 (0.58)
Possible cerebrovascular accident	1 (0.58)
Number of complications	37 (21.51)

As some patients experienced more than one complication, the sum of individual complications exceeded the total number of patients with complications.

A total of 15 patients did not undergo surgery. One of these nonsurgical patients experienced severe complications, including sepsis, septic shock, and biliary tract infection, culminating in respiratory failure. The remaining 14 nonsurgical patients did not experience any documented in-hospital complications during the study period.

### 3.3 Univariate analysis of factors influencing hospital complications in pancreatic disease patients

Using chi-square tests, t tests, and ANOVA, independent variables (study factors) were compared with the dependent variable (occurrence of hospital complications) to identify differences across study factors. The results demonstrated that disease diagnosis category (*χ*² = 8.342, *P* = 0.015), postoperative red blood cell (RBC) count (*t* = −2.552, *P* = 0.012), and postoperative haemoglobin concentration (Hb, g/L) (*t* = −2.393, *P* = 0.018) significantly influenced the occurrence of complications (*P* < 0.05) (Table 2).

**Table 2:** Univariate analysis of factors influencing hospital complications in pancreatic disease patients.

Independent variable	Complications (*n* = 37)	No complications (*n* = 135)	*P*
Sex			
Male	18 (48.65)	83 (61.48)	0.16
Female	19 (51.35)	52 (38.52)	
Age	61.89 ± 11.11	59.75 ± 10.89	0.292
BMI	22.65 ± 3.44	22.29 ± 3.53	0.581
NRS2002 scores	3.35 ± 0.54	3.17 ± 0.42	0.064
Diagnoses			
Pancreatic neoplasms	28 (75.68)	101 (74.81)	0.015
Periampullary tumour	6 (16.22)	6 (4.44)	
Cholangiocarcinoma	3 (8.10)	28 (20.75)	
Preoperative WBC (10^9^/L)	7.16 ± 5.45	5.96 ± 2.31	0.198
Preoperative RBC (10^12^/L)	4.21 ± 0.65	4.34 ± 0.65	0.308
Preoperative haemoglobin (g/L)	128.16 ± 21.17	130.99 ± 20.55	0.462
Preoperative platelet count (10^9^/L)	210.51 ± 66.29	210.62 ± 88.41	0.993
Preoperative neutrophil percentage (%)	68.52 ± 15.03	68.04 ± 11.60	0.835
Preoperative AST (U/L)	102.22 ± 127.14	63.10 ± 102.65	0.053
Preoperative ALT (U/L)	120.81 ± 150.98	88.30 ± 140.72	0.222
Preoperative ALP (U/L)	306.62 ± 354.38	202.67 ± 253.91	0.102
Preoperative total bilirubin (umol/L)	80.97 ± 111.42	52.64 ± 83.65	0.157
Preoperative direct bilirubin (umol/L)	63.58 ± 99.91	37.10 ± 71.28	0.138
Preoperative total protein (g/L)	68.59 ± 10.19	69.52 ± 7.34	0.531
Preoperative albumin (g/L)	38.25 ± 7.49	40.13 ± 5.80	0.105
Surgical methods			
Pancreatoduodenectomy	11 (29.73)	16 (11.85)	0.055
Distal pancreatectomy	5 (13.51)	21 (15.56)	
Endoscopic procedures	13 (35.14)	52 (38.52)	
Biopsy (other)	4 (10.81)	35 (25.93)	
No surgery	4 (10.81)	11 (8.14)	
Postoperative WBC (10^9^/L)	11.16 ± 4.27	10.59 ± 4.22	0.472
Postoperative RBC (10^12^/L)	3.76 ± 0.44	4.01 ± 0.57	0.012
Postoperative haemoglobin (g/L)	113.86 ± 15.63	121.55 ± 17.72	0.018
Postoperative platelet count (10^9^/L)	191.91 ± 52.83	191.30 ± 75.24	0.963
Postoperative neutrophil percentage (%)	82.16 ± 14.58	81.68 ± 11.00	0.829
Postoperative AST (U/L)	79.41 ± 76.88	89.13 ± 103.93	0.597
Postoperative ALT (U/L)	103.85 ± 91.96	102.64 ± 115.25	0.953
Postoperative ALP (U/L)	238.70 ± 259.60	179.58 ± 215.62	0.160
Postoperative total bilirubin (umol/L)	64.33 ± 73.95	52.01 ± 62.02	0.307
Postoperative direct bilirubin (umol/L)	44.51 ± 64.11	30.67 ± 51.08	0.170
Postoperative total protein (g/L)	58.48 ± 5.85	60.05 ± 6.72	0.200
Postoperative albumin (g/L)	33.74 ± 4.67	34.54 ± 4.29	0.322

Data are presented as *n* (%) or mean ± standard deviation. *P* values were derived from chi-square tests for categorical variables and independent sample t tests for continuous variables.

### 3.4 Multivariate analysis of factors influencing hospital complications in pancreatic disease patients

Through stepwise regression analysis, independent variables (study factors) were analysed against the dependent variable (occurrence of hospital complications in patients with pancreatic disease). The results indicated that the NRS2002 score significantly influenced the occurrence of hospital complications (OR = 4.20; 95% CI: 1.017–17.368; *P* = 0.047) (Table 3).

**Table 3:** Multivariate analysis of factors influencing hospital complications in pancreatic disease patients.

Independent variable	*β*	SE	OR	*P*	95% CIs
Constant	−7.702	5.460	1.990	0.158	
Sex	−0.977	0.577	0.377	0.090	0.122∼1.167
Age	−0.010	0.033	0.991	0.775	0.928∼1.057
BMI	0.034	0.072	1.035	0.631	0.899∼1.191
NRS2002 scores	1.436	0.724	4.202	0.047	1.017∼17.368
Diagnoses					
Cholangiocarcinoma					
Pancreatic neoplasms	1.225	0.934	3.403	0.190	−2.357∼432.397
Periampullary tumour	1.523	1.188	4.588	0.200	−7.236∼504.757
Preoperative WBC (10^9^/L)	−0.142	0.111	1.152	0.201	0.927∼1.432
Preoperative RBC (10^12^/L)	−0.255	1.485	0.775	0.863	0.042∼14.229
Preoperative haemoglobin (g/L)	0.068	0.054	1.070	0.207	0.963∼1.189
Preoperative platelet count (10^9^/L)	−0.008	0.006	0.992	0.163	0.981∼1.003
Preoperative neutrophil percentage (%)	0.010	0.021	1.010	0.617	0.970∼1.052
Preoperative AST (U/L)	0.003	0.007	1.003	0.677	0.990∼1.016
Preoperative ALT (U/L)	−0.003	0.005	0.997	0.495	0.987∼1.006
Preoperative ALP (U/L)	0.002	0.003	1.002	0.535	0.997∼1.006
Preoperative total bilirubin (umol/L)	−0.020	0.039	0.980	0.601	0.908∼1.058
Preoperative direct bilirubin (umol/L)	0.023	0.043	1.023	0.589	0.941∼1.113
Preoperative total protein (g/L)	0.085	0.072	1.089	0.240	0.945∼1.255
Preoperative albumin (g/L)	−0.150	0.109	0.860	0.168	0.695∼1.065
Surgical methods					
No surgery					
Pancreatoduodenectomy	1.017	0.988	2.765	0.303	−10.323∼320.152
Distal pancreatectomy	0.368	1.154	1.444	0.750	−89.492∼189.238
Endoscopic procedures	−0.248	0.903	0.781	0.784	−142.471∼99.583
Biopsy (other)	−0.827	0.996	0.437	0.406	−172.946∼77.548
Postoperative WBC (10^9^/L)	−0.015	0.092	0.985	0.866	0.823∼1.178
Postoperative RBC (10^12^/L)	−0.529	1.972	0.589	0.788	0.012∼28.109
Postoperative haemoglobin (g/L)	−0.052	0.068	0.950	0.446	0.832∼1.084
Postoperative platelet count (10^9^/L)	0.003	0.008	1.003	0.682	0.988∼1.019
Postoperative neutrophil percentage (%)	−0.008	0.023	0.992	0.734	0.949∼1.037
Postoperative AST (U/L)	0.001	0.007	1.001	0.940	0.987∼1.014
Postoperative ALT (U/L)	0.002	0.006	1.002	0.803	03990∼1.014
Postoperative ALP (U/L)	0.000	0.003	1.000	0.931	0.995∼1.006
Postoperative total bilirubin (umol/L)	−0.058	0.036	0.944	0.106	0.879∼1.013
Postoperative direct bilirubin (umol/L)	0.063	0.041	1.066	0.120	0.984∼1.154
Postoperative total protein (g/L)	−0.024	0.093	0.976	0.797	0.814∼1.171
Postoperative albumin (g/L)	0.124	0.122	1.131	0.311	0.891∼1.437

Categorical variables were modelled using dummy coding with the following reference groups: Sex: Female. Disease Diagnosis: Cholangiocarcinoma Surgical Procedure: No surgery. OR, odds ratio; CI, confidence interval.

## 4 Discussion

### 4.1 Postoperative complication profile in pancreatic disease patients

This study revealed a postoperative complication rate of 21.51% in pancreatic disease patients, which was lower than the 72.6% reported by Weinberg et al. (5). for patients undergoing distal pancreatectomy (DP). Labib et al. (6) reported infection and/or major complication rates of 11% and 16%, respectively, in 348 patients following distal pancreatectomy with splenectomy. These discrepancies may arise from differences in surgical methods, treatment protocols, or follow-up duration, as this study focused solely on complications during hospitalisation and did not track postdischarge outcomes.

The most frequent complications in this study were infection (14.53%), pancreatic fistula (7.56%), and cholangitis (3.49%). In contrast, Weinberg et al. (5). reported that endocrine derangement (25.8%), postoperative systemic inflammatory response syndrome (22.6%), and postoperative pancreatic fistula (20.9%) were the most common complications. Notably, the incidence of postoperative pancreatic fistula aligns with our findings (7).

### 4.2 Factors influencing hospital complications in pancreatic disease patients

In the univariate analysis of factors influencing hospital complications, low postoperative red blood cell (RBC) counts (*P* = 0.012) and low postoperative haemoglobin (Hb) levels (*P* = 0.018) were identified as significant risk factors. The potential mechanisms linking reduced RBC and Hb levels to complications include the following: (1) Increased infection risk: Anaemia compromises tissue oxygenation and weakens immune cell function, increasing the incidence of postoperative pneumonia, intra-abdominal infections, and other infectious complications. Studies indicate that patients with low Hb levels face a fivefold greater risk of hospital complications and increased susceptibility to infections (8, 9). (2) Delayed recovery: Low Hb levels are associated with delayed intestinal function recovery, potentially prolonging hospitalisation (10). Nakamura et al. (8). confirmed that preoperative anaemia exacerbates hospital complications in elderly patients.

According to the multivariate analysis, high NRS2002 scores (*P* = 0.047) are an independent predictor of complications. Lee et al. (11). reported that preoperative malnutrition, assessed via serum albumin levels and body mass index (BMI), was correlated with poor short-term outcomes in pancreatic head cancer patients who underwent radical pancreatoduodenectomy (PD), with a significantly greater major complication rate in the malnutrition group (36.7% vs. 21.8%, *P* = 0.032). Zhang et al. (12). reported that protein-energy malnutrition is associated with increased mortality, prolonged hospital stays, higher costs, and systemic complications in pancreatic cancer patients undergoing open PD. Gillis et al. (13). highlighted that enhanced nutritional rehabilitation reduces hospital stays by 2 days and decreases complication rates in colorectal surgery patients. Similarly, Martin et al. (14). emphasised the clinical value of preoperative immunonutrition for reducing complications and length of stay (LOS) and improving postoperative nutritional indices (e.g., the NRI and serum albumin concentration) in patients with advanced pancreatic disease. Xu et al. (15). further showed that preoperative nutritional support reduces the incidence of postoperative pancreatic fistula in high-risk patients according to the NRS2002. Conversely, preoperative frailty has also been validated as a predictor of complications in pancreatic cancer patients (16).

Owing to the limited sample size within each specific surgical procedure category (e.g., only 27 pancreatoduodenectomies), a formal subgroup analysis to identify procedure-specific risk factors was not feasible. Future larger studies are needed to explore this aspect.

### 4.3 Limitations

(1) Sample size limitations: While numerous influencing factors were explored, the relatively small sample size restricts the generalisability of the findings. Future studies with larger cohorts are needed to validate these associations. (2) Limited follow-up: This study focused solely on complications during hospitalisation. Postdischarge complications were not investigated, potentially leading to an underestimation of the true complication burden. (3) The limitations of this work are its single-centre and retrospective design. Consequently, our results require validation in larger, prospective, multicentre studies. Subsequent research should aim to definitively establish whether interventions targeting these risk factors can lead to improved clinical outcomes.

## 5 Conclusions

The postoperative complication rate in pancreatic disease patients was 21.51%, with infection (14.53%), pancreatic fistula (7.56%), and cholangitis (3.49%) being the most common complications. Low postoperative RBC counts, low postoperative haemoglobin levels, and high preoperative NRS2002 scores were significant risk factors. Future large-scale, multicentre studies are warranted to validate these findings and refine clinical strategies.

## Data Availability

The original contributions presented in the study are included in the article/Supplementary Material, further inquiries can be directed to the corresponding author.
